# An Inhibitor of Grp94 Inhibits OxLDL-Induced Autophagy and Apoptosis in VECs and Stabilized Atherosclerotic Plaques

**DOI:** 10.3389/fcvm.2021.757591

**Published:** 2021-12-06

**Authors:** Qun Wei, Hui Ren, Jun Zhang, Wen Yao, Baoxiang Zhao, Junying Miao

**Affiliations:** ^1^Shandong Provincial Key Laboratory of Animal Cells and Developmental Biology, School of Life Science, Shandong University, Qingdao, China; ^2^NHC Key Laboratory of Otorhinolaryngology (Shandong University), Department of Otorhinolaryngology, Qilu Hospital, Shandong University, Jinan, China; ^3^School of Chemistry and Chemical Engineering, Institute of Organic Chemistry, Shandong University, Jinan, China

**Keywords:** glucose-regulated protein 94, atherosclerosis, autophagy, apoptosis, adenosine monophosphate-activated protein kinase

## Abstract

**Background:** Oxidized low-density lipoprotein (oxLDL) induces vascular endothelial cell (VEC) injury and atherosclerosis through activating endoplasmic reticulum stress. Expression of glucose-regulated protein 94 (Grp94) is induced by endoplasmic reticulum stress and Grp94 is involved in cardiovascular diseases. This study aimed to determine the role of Grp94 in oxLDL-induced vascular endothelial cell injury and atherosclerosis.

**Methods and Results:** An inhibitor of Grp94, HCP1, was used to investigate the role of Grp94 in oxLDL-induced VEC injury in human umbilical vein endothelial cells and atherosclerosis in apolipoprotein E^−/−^ mice. Results showed that HCP1 inhibited autophagy and apoptosis induced by oxLDL in VECs. And we found that Grp94 might interact with adenosine monophosphate-activated protein kinase (AMPK) and activate its activity. HCP1 inhibited AMPK activity and overexpression of Grp94 blocked the effect of HCP1. Besides, HCP1 activated the activity of mechanistic target of rapamycin complex 1 (mTORC1), co-treatment with AMPK activator acadesine eliminated the effect of HCP1 on mTORC1 activity as well as autophagy. In apolipoprotein E^−/−^ mice, HCP1 suppressed autophagy and apoptosis of atherosclerotic plaque endothelium. In addition, HCP1 increased the content of collagen, smooth muscle cells, and anti-inflammatory macrophages while reducing the activity of MMP-2/9 and pro-inflammatory macrophages in the atherosclerotic lesion.

**Conclusion:** HCP1 inhibited oxLDL-induced VEC injury and promoted the stabilization of atherosclerotic plaque in apoE^−/−^ mice. Grp94 might be a potential therapeutic target in the clinical treatment of atherosclerosis.

## Introduction

As a risk factor of atherosclerosis, oxidized low-density lipoprotein (oxLDL) promotes vascular endothelial cell (VEC) injury through increasing production of reactive oxygen species (ROS), inhibition of nitric oxide release, regulation of endothelial permeability, inflammation, and apoptosis (1). We noted that oxLDL has been reported to induce autophagy in VECs (2, 3). There is substantial evidence that basal-level autophagy protects endothelial cells from oxidative damage by degrading damaged intracellular material (4). However, excessively stimulated autophagy may cause VEC death (5, 6). It's worth noting that numbers of small molecules have been reported to inhibit oxLDL-induced VEC injury through upregulation of autophagy (7–9). Recent studies revealed that downregulation of autophagy could also inhibit endothelial oxLDL-induced cell injury. High-density lipoprotein inhibited autophagy and VEC death by blocking Ca^2+^ deregulation which was caused by oxLDL (10). In a study, a butyrolactone derivative, 3BDO, was found to inhibit VEC injury induced by oxLDL through inhibition of autophagy *via* activating mechanistic target of rapamycin complex 1 (mTORC1) pathway (11). Therefore, regulation of autophagy homeostasis is vital to prevent VEC injury caused by oxLDL.

Glucose-regulated protein 94 (Grp94), localized in the endoplasmic reticulum (ER), is a member of the heat shock protein 90 (HSP90) molecular chaperone family (12). Grp94 is a canonical hallmark of ER stress whose expression is induced by unfolded protein response (13). OxLDL was reported to induce the expression of Grp94 through activating ER stress in HMEC-1 human endothelial cells and RAW264.7 macrophage cells (14, 15). ER stress can induce autophagy activation (16). OxLDL-induced ER stress is associated with autophagy activation in HMEC-1 human endothelial cells (10). Grp94 and glucose-regulated protein 78 were upregulated *via* unfolded protein response, accompanied by induction of autophagy in hypoxic conditions (17). And knockdown of glucose-regulated protein 78 was reported to block autophagy induced by ER stress (18). In addition, a component of ER stress response activating transcription factor-4 (ATF4) can regulate the transcription of LC3B (19). However, whether Grp94 is involved in oxLDL-induced autophagy and cell injury in VECs is still unknown.

Endothelial dysfunction occurs in the early stages of atherosclerosis and leads to the development and destabilization of plaque (6, 20). In particular, endothelial cell injury induced by ER stress aggravates atherosclerosis through induction of apoptosis and inflammation. Besides, apoptosis of endothelial cells, macrophages, and smooth muscle cells leads to plaque destabilization, increasing the risk of atherothrombotic disease (21). Grp94 protein level was reported to be elevated in advanced atherosclerotic plaques (22). However, the effects of Grp94 on atherosclerosis remain unclear.

Previously, we synthesized and identified a novel coumarin pyrazoline derivative HCP1 and HCP1 that was demonstrated to inhibit Grp94 activity and VEC apoptosis caused by serum and fibroblast growth factor 2 deprivation at low concentrations (23). In this study, we examined the role of Grp94 in oxLDL-induced autophagy, cell injury in VECs, and atherosclerosis in apolipoprotein E^−/−^ (apoE^−/−^) mice.

## Materials and Methods

### Cell Culture

All cell lines were maintained at 37°C under humidified conditions and 5% CO_2_. HUVECs were obtained from human umbilical cord veins as described (24). HUVECs were cultured as routine on gelatin-coated plastic dishes in M199 medium (Gibco, 31100-035) supplemented with 10% fetal bovine serum (FBS, Hyclone, SV30087.02) and 2 ng/mL fibroblast growth factor 2. All HUVECs involved in experiments were at no more than passage 10. HEK293T cells were cultured as routine in DMEM medium (Gibco, 12800–058) supplemented with 10% FBS.

### Antibodies and Reagents

Antibodies against c-Myc (sc-40), CD31 (sc-1506), α-actin (sc-32251), CD11C (sc-28671), CD206 (sc-58987), CD68 (sc-7084), normal mouse IgG (sc-2025), and horseradish peroxidase-conjugated secondary antibodies were obtained from Santa Cruz Biotechnology (Santa Cruz, CA). Antibodies of Grp94 (20292), LC3 (2775), PARP (9542), p-AMPK (2535), AMPK (5832), p-p70S6K (9205), p70S6K (9202), p-4EBP1 (9459), and 4EBP1 (9452) were from Cell Signaling Technology (USA). Antibody for His (66005-1) was purchased from Proteintech Group (USA) and the antibody of ACTB (122M4782) was from Sigma-Aldrich (USA). Secondary antibodies for immunofluorescence were goat anti-rat Alexa 488, goat anti-mouse Alexa 488, goat anti-rabbit Alexa 488, rabbit anti-goat Alexa 488, donkey anti-rabbit Alexa 546, and donkey anti-goat Alexa 546 were all from Invitrogen (USA).

HCP1 was synthesized as described (25) and dissolved in DMSO (10 mM, Sigma-Aldrich, D2650) as a stock solution. NLDL (BT-903) and oxLDL (BT-910) were purchased from Biomedical Technologies Inc. (USA). AICAR (S1802) was obtained from Selleck. cn (USA).

### Western Blot Analysis

Cells were lysed in western and IP buffer (Beyotime, P0013) containing 150 mM NaCl, 20 mM Tris-HCl (PH 7.5), 1% Triton X-100, and proteinase inhibitors mix. After centrifuging at 4°C, the supernatant was collected. Protein samples (20 μg/lane) were loaded on 15% SDS-polyacrylamide gel and then transferred to polyvinylidene difluoride (PVDF) membrane (Millipore, IPVH00010). The membranes were incubated with primary antibodies, then horseradish peroxidase-linked secondary antibodies and detected using an enhanced chemiluminescence detection kit (Thermo Fisher, 34080). The relative quantity of proteins was analyzed by Image J and normalized to loading controls.

### Cell Staining for Immunofluorescence Microscopy

Cells were fixed in 4% paraformaldehyde (w/v) for 15 min at room temperature or ice-cold methanol at −20°C for 10 min and blocked in phosphate-buffered saline (PBS), 0.01% Triton X-100 (v/v) and 5% donkey serum (v/v) for 60 min. Then cells were incubated with primary antibodies overnight at 4°C and washed in PBS three times followed by incubation with corresponding secondary antibodies for 1 h at 37°C. Fluorescence was detected by laser scanning confocal microscopy (Zeiss LSM700, Carl Zeiss Canada). ImageJ with WatershedCounting3D plug-in was used to obtain an objective number of LC3 puncta per cell. Tiff image was opened by ImageJ and the puncta count was recorded by WatershedCounting3D with parameters that allow optimal discrimination of signal/background (26). At least three images of each sample were quantitated and the experiment was repeated three times. The relative numbers of LC3 puncta per cell were normalized to the nLDL treatment group. The representative results are shown.

### Plasmids and Overexpression

The coding region of Grp94 was sub-cloned into the pCMV3-C-Myc expression vector to produce the c-Myc-Grp94-wt construct. And the construct was confirmed by DNA sequencing. The His-tagged open reading frame clone of *Homo sapiens* AMPK (CH805185) was purchased from Vigene Biosciences (USA). Cells, at 70–80% confluence, were transfected with the expression vectors for 24 h by using Lipofectamine 2000 (Invitrogen, 11668–019) following the manufacturer's instructions.

### Cell Viability Assay

Cells were plated and treated in 96-well plates followed by precipitating in 100 μl 10% trichloroacetic acid (Shenggong Biotech, Shanghai, China) for 1 h at 4°C. Then the cells were stained with 50 μl sulforhodamine B (SRB; Sigma-Aldrich, USA) for 10 min and the bounded dye was reconstituted in 100 μl of 10 mM Trisbase (pH 10.5). The optical density was read by a Spec-traMAX 190 microplate spectrophotometer (GMI Co., USA) at 540 nm. Cell viability (%) = (OD of treated group/OD of control group) × 100.

### Immunoprecipitation (IP)

Cells were washed with ice-cold phosphate-buffered saline (PBS) and lysed in IP buffer (Beyotime, P0013) containing 150 mM NaCl, 20 mM Tris–HCl (pH 7.5), 1% Triton X-100, and proteinase inhibitors mix. After centrifuging at 4°C, the supernatant was collected and pre-cleared with protein A/G agarose beads (Beyotime, P2012) for 1 h at 4°C. Then the supernatant was gathered and incubated with specific primary antibodies or normal mouse IgG (as a control) and protein A/G beads overnight at 4°C. The beads were washed three times with IP buffer and then eluted with 2 × SDS loading buffer. And immunoprecipitated proteins were detected by western blot assay.

### Hoechst 33258 Staining

Cells were stained with Hoechst 33258 (Sigma-Aldrich, USA) for 10 min at 37°C. Then we washed them twice with PBS gently and photographed them with Olympus (Japan) BH-2 fluorescence microscope. At least three images of each sample were quantitated by image J and each experiment was repeated three times. The representative results are shown.

### Animals

Male apoE^−/−^ mice (8 weeks old; C57BL/6J-knockout) used to build the atherosclerosis animal model were purchased from the Department of Laboratory Animal Science, Peking University Health Science Center (Beijing, China). The experimental design of this study was shown in Figure 5C. All procedures were under the Guide for the Care and Use of Laboratory Animals published by the US National Institutes of Health (NIH Publication No. 85–23, revised 1996). The animal experimental protocol complied with the Animal Management Rules of the Chinese Ministry of Health (document no. 55, 2001) and was approved by the Animal Care Committee of Shandong University. Mice were fed with an atherogenic high-fat diet (21% fat and 0.15% cholesterol) for 20 weeks and randomized to three groups (*n* = 6 mice/group) for treatment. The baseline group was anesthetized by isoflurane inhalation (3%) plus 1 L/min O_2_ and euthanized by exsanguination/cervical dislocation wherever appropriate. The HCP1 treatment group received 8 weeks' intraperitoneal injections of HCP1 (1 mg/kg per day). The Control group was injected with the same volume of dimethyl sulfoxide (Sigma-Aldrich, St. Louis, MO, USA) diluted with PBS.

### Bodyweight Measurement, Tissue Collection, and Organ Coefficients

The bodyweight of mice was measured once a week during HCP1 treatment. For mice at 28 and 36 weeks of age, animals were anesthetized by isoflurane inhalation (3%) plus 1 L/min O_2_ and euthanized by exsanguination/cervical dislocation wherever appropriate. The aorta and heart were rapidly extracted. The heart including aortic roots was embedded in optimal cutting temperature (OCT) embedding medium (Tissue-Tek, Torrance, CA, USA) for following histology and immunofluorescence assay. The adventitia was thoroughly stripped, and the remaining aorta was opened longitudinally and fixed in 4% paraformaldehyde for measurements of plaque surface area. Other organs including the heart, liver, spleen, lung, kidney, brain, pancreas, and thymus were removed and weighed. The organ coefficient was obtained by dividing the weight of the organ by body weight.

### Histology and Immunofluorescence

The aortic roots of mice were embedded in an OCT embedding medium and cryosections of the aortic sinus (7 μm) were prepared. Aortic root cryosections underwent Masson's trichrome staining (Sigma-Aldrich). Images of cryosections were taken with a digital camera and analyzed by ImagePro Plus. The corresponding sections were stained with primary antibodies (1:100) overnight at 4°C and incubated with the appropriate secondary antibodies (1:200) for 1 h at 37°C. Then samples were observed by laser scanning confocal microscopy (Zeiss LSM700, Carl Zeiss Canada). Six samples were detected in each group and at least three images of each sample were quantitated. Carl Zeiss AxioVision 4.6 was used to measure the fluorescence intensity of each aortic root sample. The representative results are shown.

### *In situ* Zymography

Cryosections (7 μm) of mouse aortic roots were incubated with 10 μg/ml quenched FITC-labeled DQ gelatin (Invitrogen) and 1 μg/ml propidium iodide (PI, Sigma-Aldrich) in 0.5% low melting point agarose (Invitrogen), cover-slipped, and chilled for 5 min at 4°C. Then sections were incubated at 37°C for 2 h and observed by fluorescence microscopy. Gelatinase inhibitor (MMP-2/9 inhibitor IV, Chemicon, Millipore) was added as a control. Then samples were observed by laser scanning confocal microscopy (Zeiss LSM700, Carl Zeiss Canada). Six samples were detected in each group and at least three images of each sample were quantitated. Carl Zeiss AxioVision 4.6 was used to measure the fluorescence intensity of each aortic root sample. The representative results are shown.

### En Face Aortic Arch Immunofluorescence

The fixed ascending aorta and proximal arch segment were incubated with 10% donkey serum for 30 min. Each aortic arch was then incubated simultaneously with rabbit-anti-LC3 and goat-anti-CD31 overnight at 4°C and incubated with the appropriate secondary antibodies: rabbit anti-goat Alexa 488 and donkey anti-rabbit Alexa 546 for 1 h at 37°C. The nuclei were stained with DAPI. Then aorta segments were pinned flat with the endothelium facing up on glass slides, and LC3 patches were detected by laser scanning confocal microscopy (Zeiss LSM700, Carl Zeiss Canada). At least three images of each sample were quantitated by image J and six samples were detected in each group. The representative results are shown.

### Apoptosis in Atherosclerotic Plaque

Apoptotic cells in the atherosclerotic lesion were detected by TUNEL using an *in situ* cell death detection kit (Sigma, 12156792910) according to the manufacturer's instructions and sections were counterstained with DAPI to detect nuclei. Then samples were detected by laser scanning confocal microscopy (Zeiss LSM700, Carl Zeiss Canada). At least three images of each sample were quantitated by image J and six samples were detected in each group. The representative results are shown.

### Statistical Analyses

Results are reported as mean ± SEM. We used sample sizes of 6 mice per group (α = 0.05). All experiments were repeated at least three times independently. For statistical analysis, Graph Pad Prism software (version 5.0) was used. Statistical comparisons for data were performed using student's *t*-test and one-way analysis of variance (ANOVA) followed by Tukey: compare all pairs of columns test. Differences were regarded as statistically significant when *p* < 0.05.

## Results

### HCP1 Inhibited OxLDL-Induced HUVEC Injury

**Figure 1:**
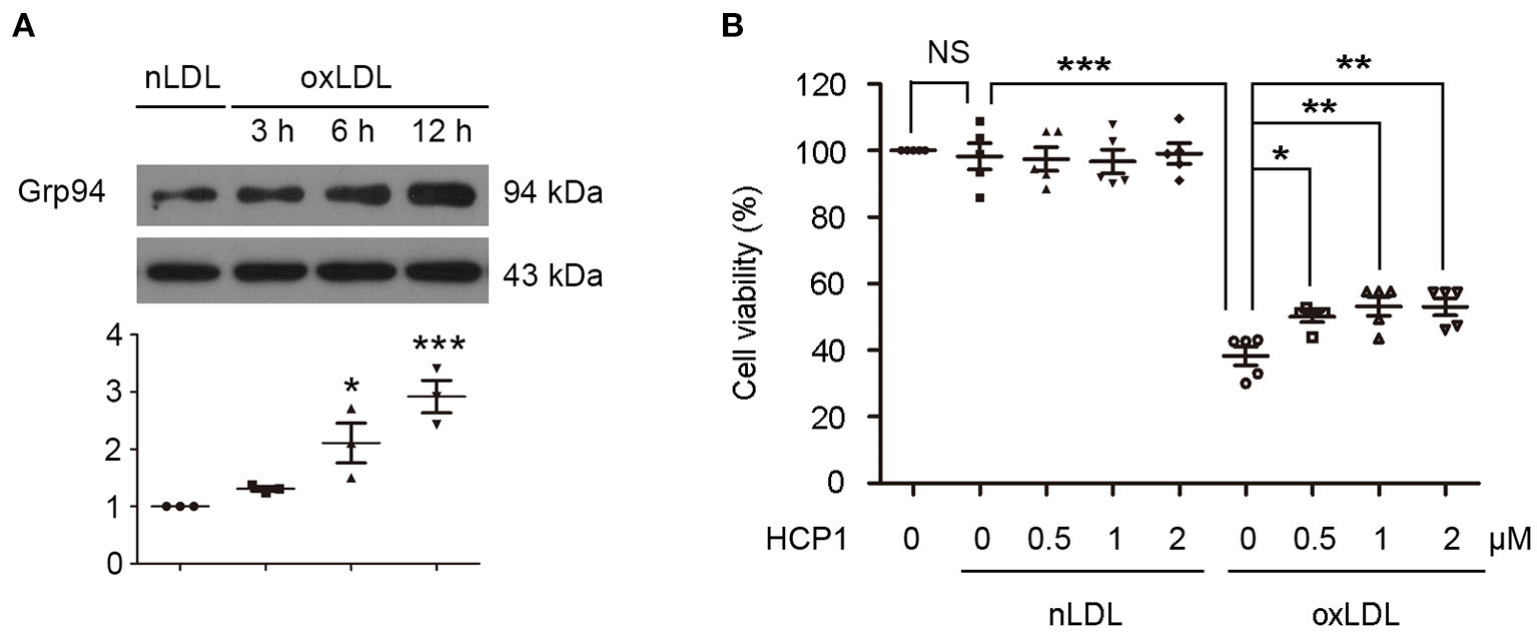
OxLDL upregulated Grp94 level and HCP1 inhibited oxLDL-induced viability reduction in HUVECs. **(A)** Protein levels of Grp94 were detected after treatment with nLDL (50 μg/ml) or oxLDL (50 μg/ml) for the indicated time in HUVECs. Data are mean ± SEM; *p < 0.05, ***p < 0.001; *n* = 3. **(B)** Viability of HUVECs treated with nLDL (50 μg/ml) or oxLDL (50 μg/ml) with or without HCP1 (0.5, 1, 2 μM) for 48 h. Data are mean ± SEM; *p < 0.05, **p < 0.01, ***p < 0.001, NS *p* > 0.05; *n* = 5. Statistical analyses were performed using one-way ANOVA.

Firstly, we detected the effect of HCP1 on oxLDL-induced VEC injury. Western blot analyses showed that oxLDL increased Grp94 protein level in human umbilical vein endothelial cells (HUVECs) (Figure 1A). Treatment with HCP1 inhibited cell viability reduction induced by oxLDL in HUVECs (Figure 1B). These results suggested that HCP1 suppressed oxLDL-induced HUVEC injury.

### HCP1 Suppressed OxLDL-Induced Autophagy in HUVECs

**Figure 2:**
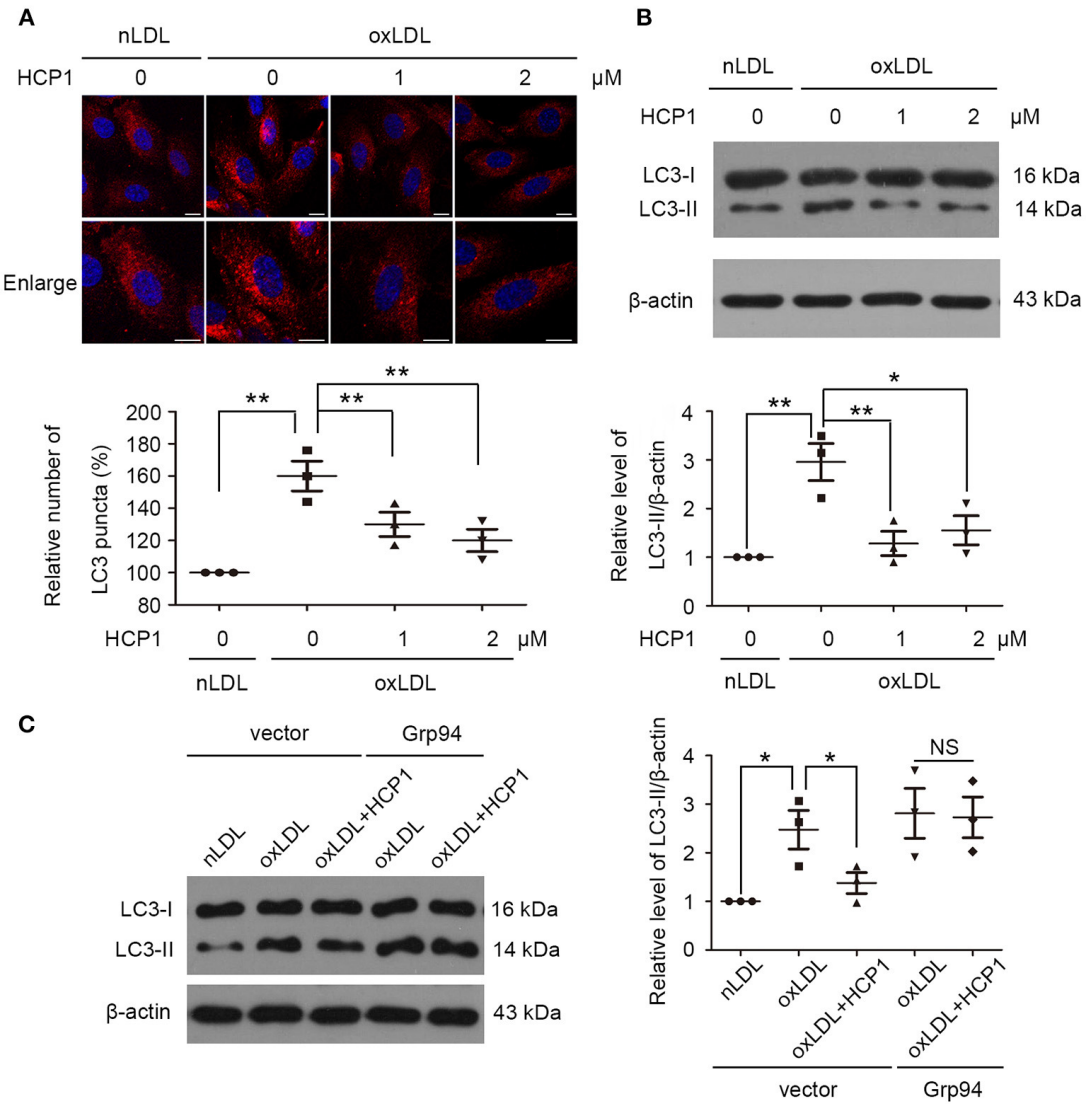
HCP1 suppressed HUVEC autophagy induced by oxLDL. **(A)** Immunofluorescence staining of LC3 was performed after treatment with nLDL (50 μg/ml), oxLDL (50 μg/ml) with or without HCP1 (1, 2 μM) for 12 h in HUVECs. Bar charts show the quantification of average endogenous LC3 puncta per cell. Scale bar, 10 μm. **(B)** Protein levels of LC3-II were detected after treatment with nLDL (50 μg/ml), oxLDL (50 μg/ml) with or without HCP1 (1, 2 μM) for 12 h in HUVECs. **(C)** Protein levels of LC3-II in HUVECs transfected with c-Myc empty vector or c-Myc-Grp94-wt plasmid for 24 h were detected after treatment with nLDL (50 μg/ml), oxLDL (50 μg/ml) with or without HCP1 (1 μM) for 12 h. Data are mean ± SEM; *p < 0.05, **p < 0.01, NS *p* > 0.05; *n* = 3. Statistical analyses were performed using one-way ANOVA.

Then we examined the effect of HCP1 on oxLDL-induced autophagy in HUVECs. Microtubule-associated protein 1 light chain 3 (MAP1LC3/LC3) is a ubiquitin-like protein involved in the formation of autophagosomes. The PE-conjugated form LC3-II is the only protein marker that is reliably associated with completed autophagosomes as well as phagophores. Therefore, LC3 is referred to as an autophagosome marker (27). Treatment with HCP1 (1 μM, 2 μM) decreased the number of LC3 dots per cell (Figure 2A) as well as the protein level of LC3-II (Figure 2B). Furthermore, transfection with c-Myc-Grp94-wt plasmid blocked the effect of HCP1 compared with the empty vector-transfected groups (Figure 2C). These results suggested that inhibition of Grp94 by HCP1 suppressed oxLDL-induced autophagy in HUVECs.

### Grp94 Might Interact With AMPK and Activate Its Activity

OxLDL induced ROS generation and autophagy in HUVECs and the autophagic response was reported to be mediated by the ROS-LOX-1 pathway (4). ROS could activate adenosine monophosphate-activated protein kinase (AMPK) through two key upstream kinases calcium-dependent protein kinase 2 or serine threonine kinase 11 (28, 29). In addition, HSP90 was found to interact with AMPK and maintain its AMP-activated kinase activity (30).

**Figure 3:**
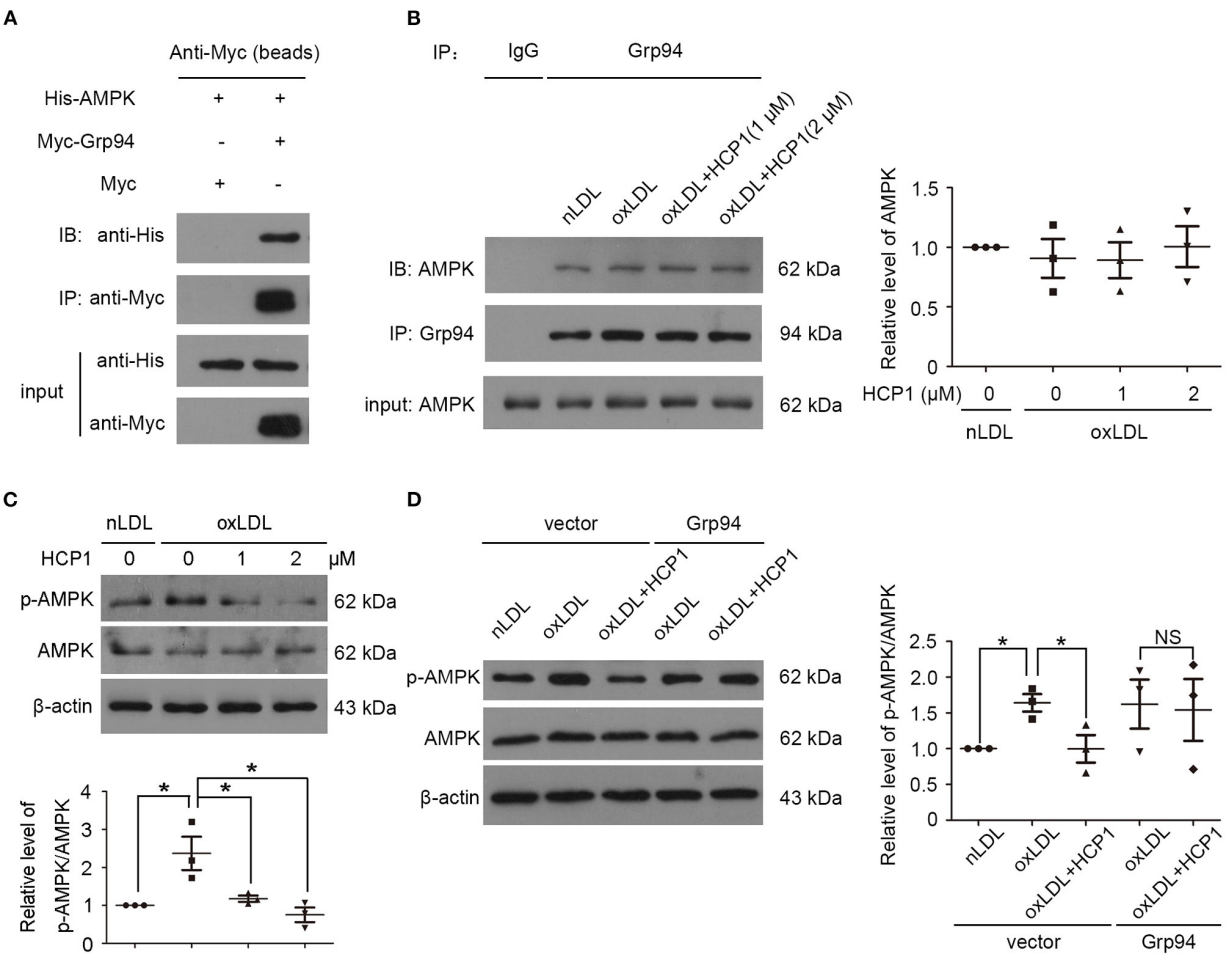
Grp94 might interact with AMPK and activate its activity. **(A)** Co-immunoprecipitation (Co-IP) of His-tagged AMPK proteins with Myc-tagged Grp94 from HEK293T cells transfected His-AMPK with Myc (lane 1) or Myc-Grp94 (lane 2). The lower panel showed expression levels of His-AMPK and Myc-Grp94 in the experiment. **(B)** Co-IP of AMPK with Grp94 from HUVECs treated with nLDL (50 μg/ml), oxLDL (50 μg/ml) with or without HCP1 (1, 2 μM) for 3 h. Co-immunoprecipitated AMPK was quantified in immunoblot with anti-AMPK antibody in western blot. **(C)** Protein levels of phosphorylated AMPKα (p-AMPKα, Thr172) and total AMPKα in HUVECs treated with nLDL (50 μg/ml), oxLDL (50 μg/ml) with or without HCP1 (1, 2 μM) for 6 h. Bar charts show quantification of the ratio of p-AMPKα to total AMPKα. **(D)** Protein levels of phosphorylated AMPKα (p-AMPKα, Thr172) and total AMPKα in HUVECs transfected with c-Myc empty vector or c-Myc-Grp94-wt plasmid for 24 h were detected after treatment with nLDL (50 μg/ml), oxLDL (50 μg/ml) with or without HCP1 (1 μM) for 6 h. Data are mean ± SEM; *p < 0.05, NS *p* > 0.05; *n* = 3. Statistical analyses were performed using one-way ANOVA.

Therefore, we detected whether Grp94 could interact with AMPK and influence its activity. The interaction between Grp94 and AMPK was detected in HEK293T cells and HUVECs by co-immunoprecipitation experiments. Results showed that Grp94 might interact with AMPK and treatment with HCP1 did not influence the interaction between them (Figures 3A,B). AMPK is activated by the phosphorylation of Thr172 in the α subunit (31). Treatment with HCP1 inhibited AMPK activity by decreasing the phosphorylation of AMPK (Thr172) in HUVECs (Figure 3C). Furthermore, transfection with c-Myc-Grp94-wt plasmid blocked the effect of HCP1 on AMPK activity compared with the empty vector-transfected groups (Figure 3D). These results suggested that inhibition of Grp94 by HCP1 suppressed AMPK activity in HUVECs.

### HCP1 Suppressed Autophagy Induced by OxLDL Dependent on AMPK-MTORC1 Pathway

**Figure 4:**
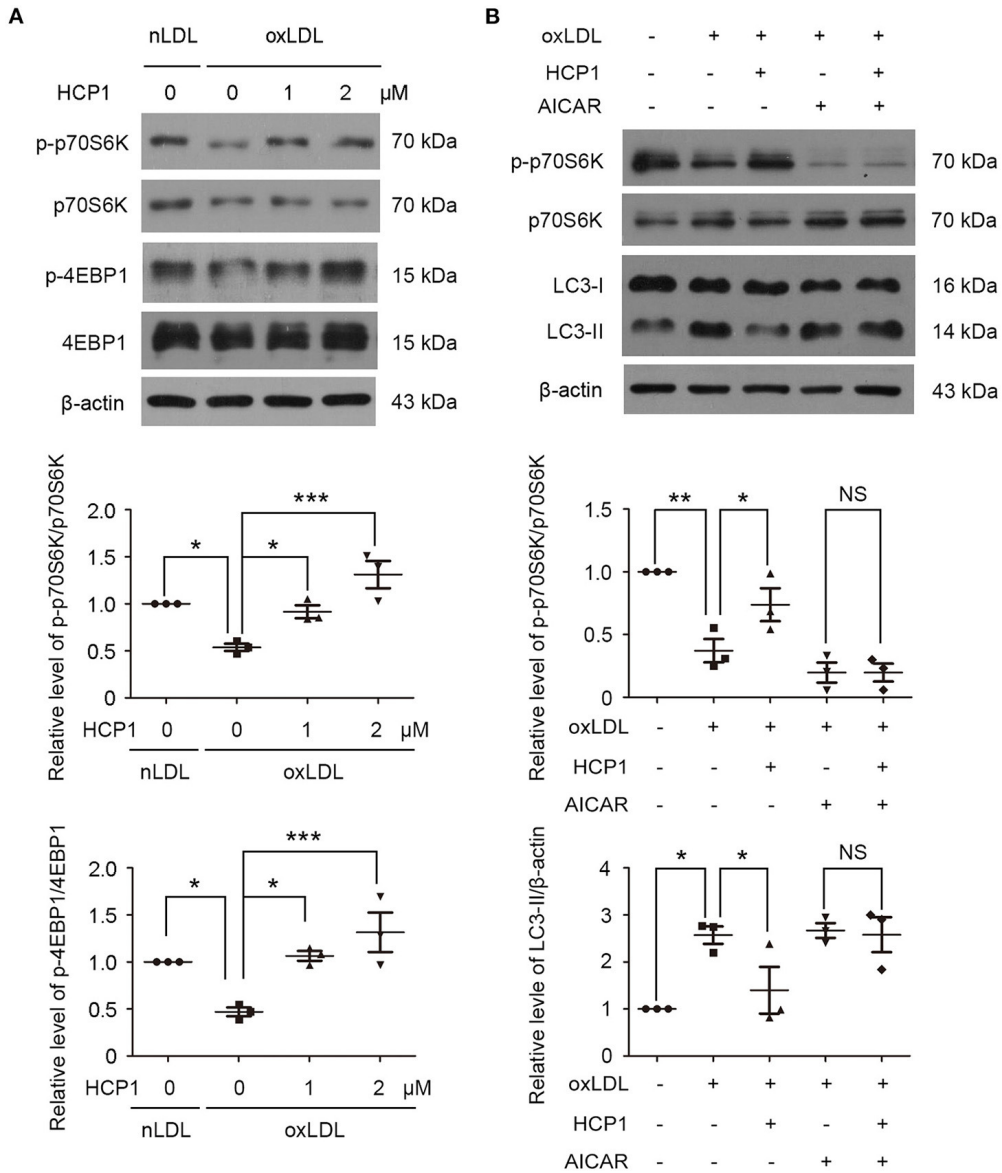
HCP1 activated mTORC1 activity and reduced LC3-II protein level through inhibiting AMPK. **(A)** Protein levels of phosphorylated p70S6K (p-p70S6K, Thr389), total p70S6K, phosphorylated 4EBP1 (p-4EBP1, Thr37/46), and total 4EBP1 in HUVECs treated with nLDL (50 μg/ml), oxLDL (50 μg/ml) with or without HCP1 (1, 2 μM) for 12 h. Bar charts show quantification of the ratios of p-p70S6K to total p70S6K and p-4EBP1 to total 4EBP1. **(B)** Protein levels of phosphorylated p70S6K (p-p70S6K, Thr389), total p70S6K, and LC3-II in HUVECs treated with nLDL (50 μg/ml), oxLDL (50 μg/ml) with or without HCP1 (1 μM), AICAR (5 mM) for 12 h. Bar charts show quantification of the ratios of p-p70S6K to total p70S6K and protein levels of LC3-II. Data are mean ± SEM; *p < 0.05, **p < 0.01, ***p < 0.001, NS *p* > 0.05; *n* = 3. Statistical analyses were performed using one-way ANOVA.

MTORC1 is a master negative regulator of autophagy and AMPK inhibits mTORC1 activity by phosphorylating TSC2 and RAPTOR (32). In accordance with previous studies, we found that treatment with oxLDL inhibited mTORC1 activity and reduced the phosphorylation of mTORC1 downstream targets ribosomal protein S6 kinase (p70S6K) and 4E-binding protein 1 (4EBP1) (11). Therefore, we detected the effects of HCP1 on mTORC1 activity. Treatment with HCP1 elevated the phosphorylation of p70S6K and 4EBP1 indicated that HCP1 activated mTORC1 activity (Figure 4A). Furthermore, co-treatment with AMPK activator acadesine (AICAR) eliminated the effect of HCP1 on mTORC1 activity suggested that HCP1 inhibited the AMPK-mTORC1 pathway. In addition, AICAR also inhibited the effect of HCP1 on protein levels of LC3-II (Figure 4B). These results demonstrated that HCP1 inhibited oxLDL-induced autophagy dependent on the AMPK-mTORC1 pathway.

### HCP1 Suppressed OxLDL-Induced Apoptosis in HUVECs

**Figure 5:**
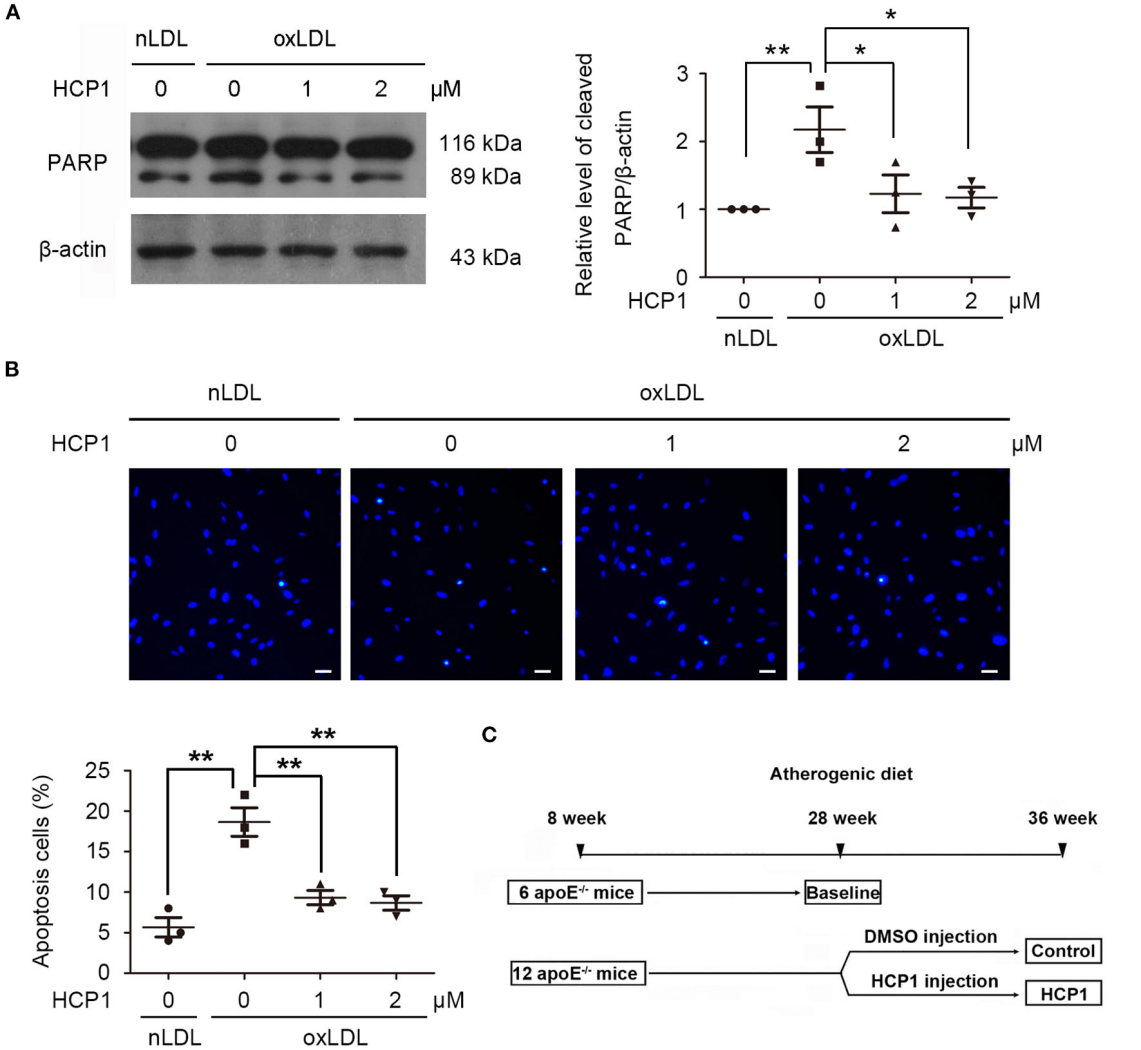
HCP1 suppressed HUVEC apoptosis induced by oxLDL. **(A)** Protein levels of cleaved PARP were detected after treatment with nLDL (50 μg/ml), oxLDL (50 μg/ml) with or without HCP1 (1, 2 μM) for 48 h in HUVECs. **(B)** Hoechst 33258 staining and quantification of apoptotic HUVECs after treatment with nLDL (50 μg/ml), oxLDL (50 μg/ml) with or without HCP1 (1, 2 μM) for 48 h. Scale bar, 20 μm. **(C)** Design of *in vivo* experiment. Eight-week-old apoE^−/−^ mice were fed with an atherogenic diet for 20 weeks and randomized to three groups (*n* = 6 mice/group) for treatment. The baseline group was euthanized. The HCP1 treatment group received 8 weeks' intraperitoneal injections of HCP1 (1 mg/kg per day) and the control group was injected with the same volume of DMSO diluted with PBS. Data are mean ± SEM; *p < 0.05, **p < 0.01; *n* = 3. Statistical analyses were performed using one-way ANOVA.

Excessive activation of autophagy can lead to apoptosis (33). The effects of HCP1 on oxLDL-induced apoptosis were determined. Poly (ADP-ribose) Polymerase (PARP) is a family of 17 proteins and PARP1 is the most extensively studied one. PARP1 participates in several cell stress processes, especially DNA damage repair. It can bind to DNA signal- and double-strand breaks, then parylate histones and other DNA repair proteins as part of the DNA repair mechanism. During apoptosis, caspases cleave PARP1 and separate the catalytic domain from the DNA binding domain. PARP1 becomes inactive and loses its ability to respond to DNA damage, resulting in cell death (34). HCP1 reduced protein levels of cleaved PARP (Figure 5A) and apoptosis rate (Figure 5B) in HUVECs indicated that HCP1 suppressed oxLDL-induced apoptosis.

### HCP1 Inhibited Autophagy and Apoptosis in the Endothelium of ApoE^–/–^ Mice

**Figure 6:**
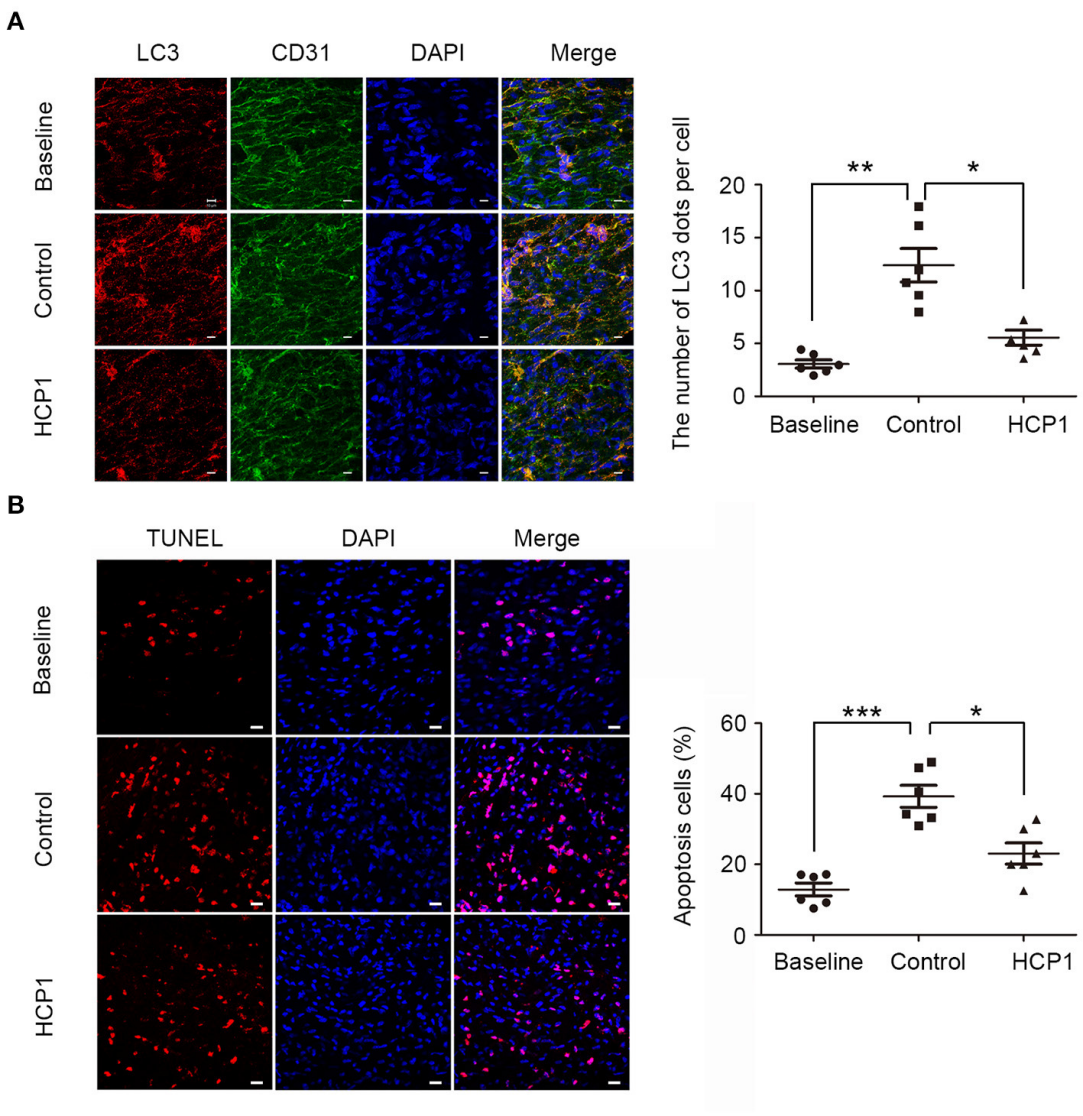
HCP1 inhibited endothelium autophagy and apoptosis in apoE^−/−^ mice. **(A)** *En face* staining of LC3 patches in the thoracic aorta endothelium of apoE^−/−^ mice. Endothelial cells were marked with CD31. Nuclei were stained with DAPI. Bar charts show the quantification of average endogenous LC3 dots per cell. Scale bar, 10 μm. **(B)** *En face* TUNEL staining of apoptotic cells in the thoracic aorta endothelium of apoE^−/−^ mice. Nuclei were stained with DAPI. Bar charts show the quantification of the percentage of apoptotic cells. Scale bar, 20 μm. Data are mean ± SEM; *p < 0.05, **p < 0.01, ***p < 0.001; *n* = 6. Statistical analyses were performed using one-way ANOVA.

Endothelial dysfunction is closely associated with atherosclerosis. To further elucidate the role of Grp94 in atherosclerosis, we examined the effect of HCP1 on the atherosclerotic plaque in apoE^−/−^ mice (Figure 5C). Firstly, we assessed the level of autophagy in the endothelium of advanced atherosclerotic lesions by *en face* immunofluorescent staining of LC3 dots. Results showed that the number of LC3 puncta per cell was significantly decreased in the HCP1 treatment group (Figure 6A), suggesting that HCP1 inhibited autophagy of atherosclerotic vascular endothelium in apoE^−/−^ mice. In addition, we detected and quantified apoptosis in the endothelium of plaque by terminal deoxynucleotidyl transferase-mediated dUTP nick-end labeling (TUNEL) staining. Results showed that treatment with HCP1 inhibited vascular endothelium apoptosis in plaque (Figure 6B). Therefore, HCP1 suppressed autophagy and apoptosis in the endothelium of apoE^−/−^ mice.

### HCP1 Stabilized Established Atherosclerotic Lesion in ApoE^–/–^ Mice

**Figure 7:**
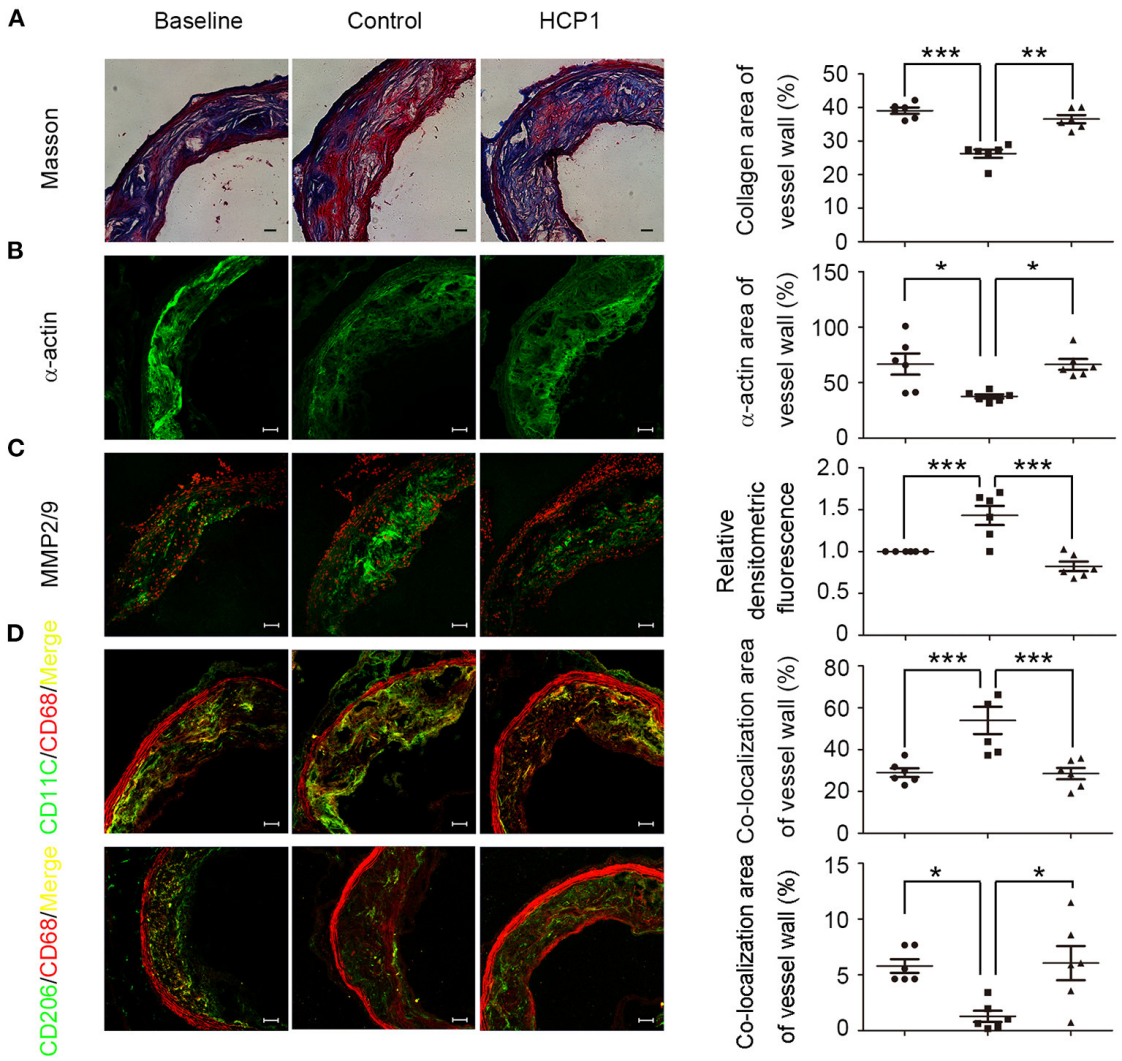
HCP1 improved aortic atherosclerotic plaque stabilization in apoE^−/−^ mice. From the top to the bottom panel, masson trichrome staining of collagen (in blue) **(A)**, immunostaining for mouse α-smooth muscle actin **(B)**, *in situ* zymography detecting MMP-2/9 activity **(C)**, double-stained images of co-localization (yellow) of CD11C (green) and CD68 (red) -positive areas **(D)** and double-stained images of co-localization (yellow) of CD206 (green), and CD68 (red) -positive areas **(D)**. Scale bars, 50 μm. Bar charts show quantification of collagen, α-actin area in the atherosclerotic lesion, MMP-2/9 activity, weighted colocalization coefficients for CD11C and CD206 positive areas in baseline, control, and HCP1-treated groups. Data are mean ± SEM; *p < 0.05, **p < 0.01, ***p < 0.001; *n* = 6. Statistical analyses were performed using one-way ANOVA.

Unstable plaques are typically presented with a large necrotic core covered by a thin fibrous cap and a highly inflammatory cell content (21). Endothelial cell, macrophage, and smooth muscle cell apoptosis in plaque contributes to the expansion of necrotic core. Treatment with HCP1 inhibited cell apoptosis of the whole plaque including endothelial cells, macrophages, and smooth muscle cells (Supplementary Figure 1). Smooth muscle cells are predominant in the fibrous cap and thicken atherosclerotic lesions by producing collagen, elastin, and other matrix components. Apoptosis of smooth muscle cells in the fibrous cap reduces extracellular matrix protein production and leads to thinner fibrous caps. Compared with the control group, mice treated with HCP1 showed increasing plaque collagen content and smooth muscle cells (Figures 7A,B). The proteolytic enzymes matrix metalloproteinases play important roles in weakening the fibrous cap and promoting plaque rupture. HCP1 treatment reduced the activity of MMP-2/9 of the atherosclerotic lesion in apoE^−/−^ mice (Figure 7C). Smooth muscle cell apoptosis also exacerbates plaque inflammation and the inflammatory macrophages can induce smooth muscle cell apoptosis. Treatment with HCP1 decreased pro-inflammatory macrophages known as M1 while increased anti-inflammatory macrophages known as M2 (Figure 7D) (35). Therefore, HCP1 promoted the stabilization of atherosclerotic lesion.

To determine whether there is potential toxicity of HCP1 to apoE^−/−^ mice, toxicity experiments were carried out. We evaluated the influence of HCP1 on body weight and organ coefficients including heart, liver, spleen, lung, kidney, brain, pancreas, and thymus. The results showed that there were no significant differences in body weight and organ coefficients between the control group and HCP1-treated group (Supplementary Figure 2).

## Discussion

**Figure 8:**
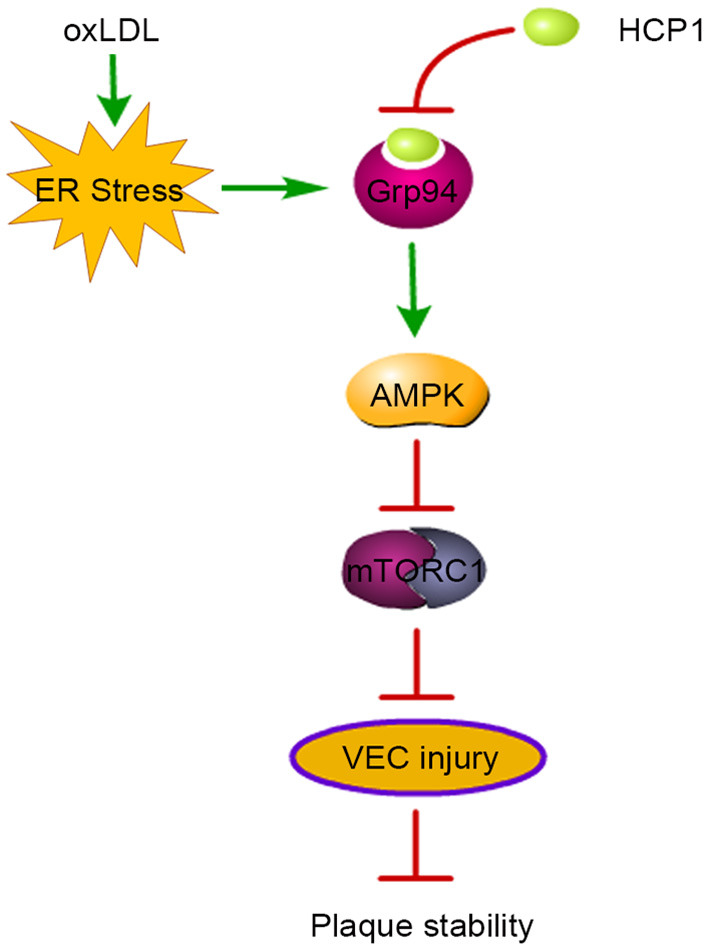
Schematic diagram of HCP1-induced plaque stability in atherosclerosis. OxLDL induced Grp94 upregulation through ER stress and autophagy which leads to VEC injury and plaque instability in apoE^−/−^ mice. Grp94 inhibitor HCP1 suppressed oxLDL-induced autophagy through inhibition of the AMPK-mTORC1 pathway. Thus, HCP1 inhibited oxLDL-induced VEC injury and promoted plaque stability in apoE^−/−^ mice.

In this study, we found that HCP1 suppressed oxLDL-induced VEC autophagy dependent on Grp94-AMPK-mTORC1 pathway. And HCP1 inhibited oxLDL-induced VEC viability reduction, apoptosis and promoted the stabilization of atherosclerotic plaque in apoE^−/−^ mice (Figure 8).

Autophagy can be induced by starvation and can promote cell survival in many cell types. However, VECs are directly in contact with serum *in vivo* and starvation induces apoptosis rapidly, giving rise to many cardiovascular diseases (36). Therefore, it is necessary to study the mechanism of autophagy in VECs in the presence of serum and FGF-2. HCP1 inhibited autophagy in VECs in the presence of serum and FGF-2, which provided us with a powerful tool for studying autophagy in VECs under normal culture conditions.

As an essential master chaperone in ER, Grp94 is involved in quality control of client proteins, regulation of Ca^2+^ homeostasis, and immune response (37). Inhibition of Grp94 by HCP1 suppressed oxLDL-induced VEC autophagy indicated that Grp94 also participated in the regulation of autophagy. Grp94 has been found to possess ATPase activity which could bind and hydrolyze ATP (38). AMPK is a heterotrimeric complex comprising catalytic α-subunit, regulatory β-subunit, and γ-subunit (39). ATP, ADP, and AMP competitively bind to the γ-subunit of AMPK. Under energy stress, AMP and/or adenosine diphosphate bind to regulatory γ-subunit and activate the kinase through increasing phosphorylation of Thr172 by upstream kinases, inhibiting Thr172 dephosphorylation by protein phosphatases and allosteric activation (31). Besides, phosphorylation of AMPK in α-subunit other than Thr172 is reported to inhibit AMPK activity (40, 41). Protein-protein interactions and subcellular distribution are also related to AMPK activity (42). HCP1 inhibited Grp94 and decreased phosphorylation of Thr172 in α-subunit of AMPK which suppressed AMPK activity. Therefore, we speculated that Grp94 might positively regulate AMPK activity in the following ways. Firstly, Grp94 might phosphorylate AMPK at Thr172 directly through its ATPase activity. Besides, the interaction between Grp94 and AMPK might also regulate AMPK activity. In addition, HCP1 might block the binding of ATP to Grp94 and thus promote the binding of ATP and AMPK which causes AMPK inhibition indirectly.

OxLDL induces constant ER stress which promotes apoptosis (43). ER stress induced autophagy and apoptosis through many common upstream signaling pathways including PERK/ATF4, IRE1α, ATF6, and Ca^2+^. Furthermore, there are dual directional regulation mechanisms between autophagy and apoptosis. Generally, autophagy inhibits apoptosis by clearing unfolded/misfolded proteins or damaged organelles, suppressing caspase-8 activation, and eliminating SQSTM1/p62 to protect cells suffering ER stress. However, in some cases, autophagy can induce apoptosis through activating caspase-8 activity and degrading inhibitors of apoptosis (IAPs), which aggravates cell injury induced by ER stress. In addition, activation of apoptosis-related proteins can inhibit autophagy *via* cleaving autophagy-related proteins, including Beclin-1, Atg4D, Atg3, and Atg5 (33). In our previous study, we found inhibition of oxLDL-induced autophagy by 3BDO, an activator of mTOR, could suppress apoptosis in VECs (11). Therefore, HCP1 might suppress oxLDL-induced apoptosis through inhibition of autophagy in HUVECs. However, the relationship between Grp94-mediated apoptosis and autophagy needs further investigation.

Grp94 is closely associated with the pathogenesis of tumors and glaucoma, which emerges as a promising therapeutic target in clinical treatment (37, 44). As a risk factor of atherosclerosis, oxLDL not only participates in the development of atherosclerosis but also contributes to diabetes mellitus and several autoimmune diseases (45). Inhibition of oxLDL-induced endothelial injury may be a valuable therapeutic strategy for ameliorating plaque instability. We found that HCP1 inhibited oxLDL-induced endothelial injury and promoted lesion stability in apoE^−/−^ mice, suggesting that Grp94 might be detrimental to atherosclerotic plaque stabilization. These support Grp94 as a potential therapeutic target in the treatment of atherosclerosis. However, as an essential master chaperone of the HSP90 protein family, off-target in Grp94 inhibition might cause severe side effects (37). Therefore, Grp94 selective inhibitors are urgently needed and might be promising anti-atherosclerotic drugs.

## Data Availability Statement

The original contributions presented in the study are included in the article/Supplementary Material, further inquiries can be directed to the corresponding author.

## Ethics Statement

The animal study was reviewed and approved by Animal Care Committee of Shandong University.

## Author Contributions

QW and JM designed the study, interpreted data, and wrote the manuscript. QW, HR, JZ, WY, and BZ performed laboratory measurements and analyzed data. All authors contributed to the article and approved the submitted version.

## Funding

This work was supported by the National Natural Science Foundation of China (Nos. 31870831, 31871407, 31741083, and 91539105).

## Conflict of Interest

The authors declare that the research was conducted in the absence of any commercial or financial relationships that could be construed as a potential conflict of interest.

## Publisher's Note

All claims expressed in this article are solely those of the authors and do not necessarily represent those of their affiliated organizations, or those of the publisher, the editors and the reviewers. Any product that may be evaluated in this article, or claim that may be made by its manufacturer, is not guaranteed or endorsed by the publisher.
