# Supplementary material for: An Inhibitor of Grp94 Inhibits OxLDL-Induced Autophagy and Apoptosis in VECs and Stabilized Atherosclerotic Plaques
Source: Front Cardiovasc Med. 2021 Dec 6;8:757591. doi: 10.3389/fcvm.2021.757591 (PMC8687133; doi:10.3389/fcvm.2021.757591)
Supplement: Supplementary file 1 [file Data_Sheet_1.pdf]

Supplementary Material

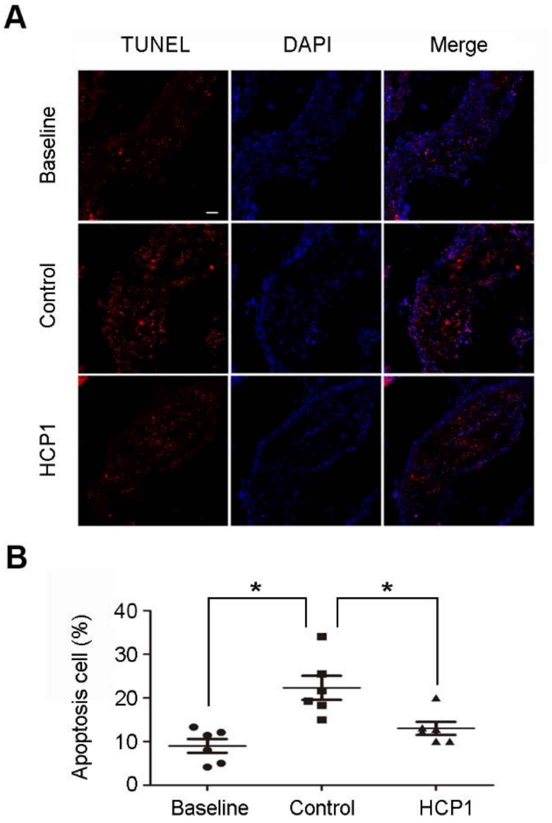

Supplementary Figure 1. HCP1 inhibited apoptosis in aortic root lesions of apoE$^{-/-}$ mice. (A) Sections from the mice aortic roots were labeled by TUNEL to detect apoptotic cells and counterstained with DAPI to detect nuclei. Scale bar, 50 µm. (B) Quantification of TUNEL-positive

cells in the plaque area. Data are mean ± SEM; *$p < 0.05$; n=6. Statistical analyses were performed using one-way ANOVA.

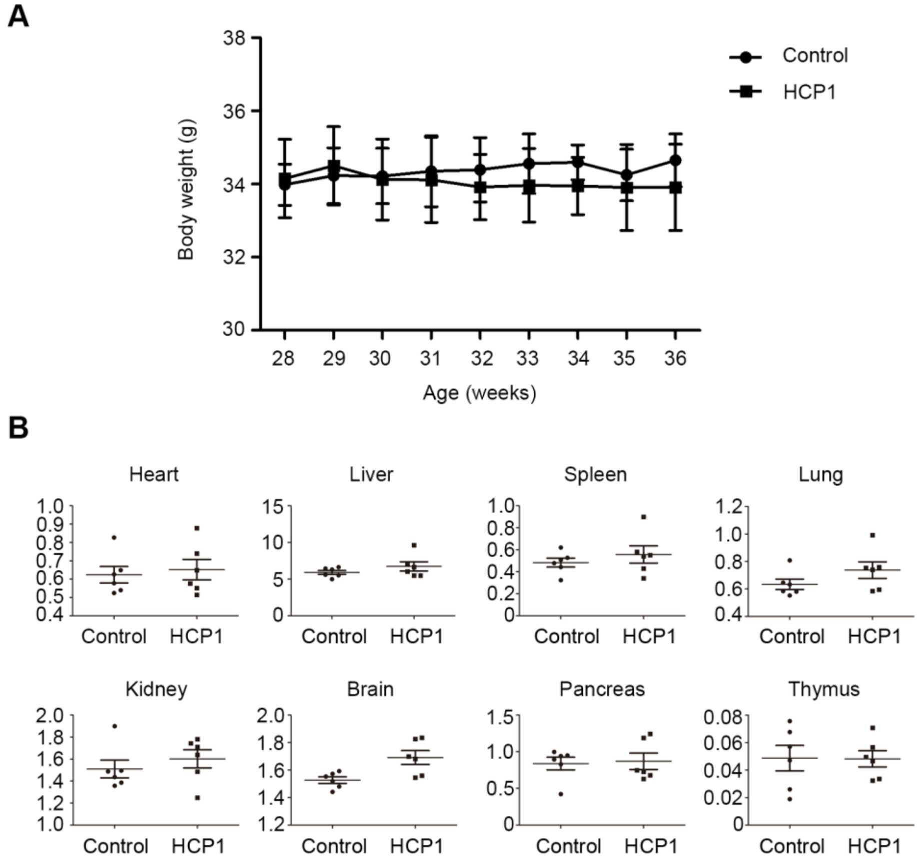

Supplementary Figure 2. Effects of HCP1 on body weight and organ coefficients in apoE$^{-/-}$ mice. (A) Body weight was measured every week during intraperitoneal injection period. (B) Organ coefficients of apoE$^{-/-}$mice. Data are mean ± SEM; n=6. Statistical analyses were performed using a two-tailed unpaired t-test.
